# Dynamic Mechanical Properties and Microstructure of Graphene Oxide Nanosheets Reinforced Cement Composites

**DOI:** 10.3390/nano7120407

**Published:** 2017-11-24

**Authors:** Wu-Jian Long, Jing-Jie Wei, Hongyan Ma, Feng Xing

**Affiliations:** 1 Guangdong Provincial Key Laboratory of Durability for Marine Civil Engineering, Shenzhen Durability Center for Civil Engineering, College of Civil Engineering, Shenzhen University, Shenzhen 518060, Guangdong, China; 2150150416@email.szu.edu.cn (J.-J.W.); xingf@szu.edu.cn (F.X.); 2 Department of Civil, Architectural and Environmental Engineering, Missouri University of Science and Technology, Rolla, MO 65401, USA; mahon@mst.edu

**Keywords:** graphene oxide (GO), cement composite, dynamic mechanical properties, microstructures, strength

## Abstract

This paper presents an experimental investigation on the effect of uniformly dispersed graphene oxide (GO) nanosheets on dynamic mechanical properties of cement based composites prepared with recycled fine aggregate (RFA). Three different amounts of GO, 0.05%, 0.10%, and 0.20% in mass of cement, were used in the experiments. The visual inspections of GO nanosheets were also carried out after ultrasonication by transmission electron microscope (TEM) atomic force microscope (AFM), and Raman to characterize the dispersion effect of graphite oxide. Dynamic mechanical analyzer test showed that the maximum increased amount of loss factor and storage modulus, energy absorption was 125%, 53%, and 200% when compared to the control sample, respectively. The flexural and compressive strengths of GO-mortar increased up to 22% to 41.3% and 16.2% to 16.4% with 0.20 wt % GO at 14 and 28 days, respectively. However the workability decreased by 7.5% to 18.8% with 0.05% and 0.2% GO addition. Microstructural analysis with environmental scanning electron microscopy (ESEM)/backscattered mode (BSEM) showed that the GO-cement composites had a much denser structure and better crystallized hydration products, meanwhile mercury intrusion porosimetry (MIP) testing and image analysis demonstrated that the incorporation of GO in the composites can help in refining capillary pore structure and reducing the air voids content.

## 1. Introduction

The rapid economic growth in China has encouraged an enormous number of construction activities. Construction and demolition (C&D) wastes that are produced during new construction, renovation, and demolition of buildings and structures have become a serious problem in many countries [1]. Numerous studies have investigated the effects and impacts of C&D wastes on the environment, economy, and society [2,3]. In many countries, a large amount of the recycled aggregate is derived from this waste. Owing to the increasing cost of landfill, the scarcity of natural resources coupled with the greater demand for aggregates in construction and the use of recycled aggregate to partially or totally replace the natural aggregate has, therefore, become a common practice.

So far, most researches on recycled aggregate have focused on the mechanical properties, durability, and damping capability of recycled fine aggregate (RFA) mortar or recycled aggregate concrete. A number of publications on the use of RFA in mortar or concrete concluded that strength and durability decreased with the increase of the replacement ratio of RFA [4,5,6,7]. According to Kim and Yun [8], the recycled concrete including lower RFA grade showed clear decreases in bond strength with an increasing RFA ratio. Moreover, the porosity of mortar containing recycled fine aggregate is normally larger than that of the mortar made with natural sand [9,10]. This is attributed to the large surface area and poor surface quality of the recycled fine aggregate, which create a weak interface transition zone (ITZ), and thus decreases bonding between the matrix and the fine aggregate [11,12].

In addition, a series of studies on improving the damping capability and stiffness of the cement paste have been conducted since 1996 by Fu et al. [13] and Xu et al. [14,15]. The enhancement of several admixtures on damping capability of cement paste was obtained at 30 °C to 120 °C in these studies. According to Liew et al. [16] and Long et al. [17], the damping of cement composites was affected not only by the stress amplitude, but also by factors such as the cross-section shapes, load types, and loading positions. The dynamic behavior of recycled- rubber sand mixtures was studied by Li et al. [18]. Their results demonstrated that the mix ratio significantly affected the dynamic modulus.

Currently, the applications of nanotechnology have been gaining popularity in different fields of science and technology [19,20]. The use of nano materials in concrete is more popular for its better properties shown in the fresh and hardened states of concrete due to their high specific surface area. The typical materials that are used with nano size are nano-SiO_2_, nano-TiO_2_, nano-Fe_2_O_3_, nano-Al_2_O_3_, carbon nanotubes/fibers, and graphene oxide (GO) nanosheets. The use of nano particles in cement based products has been increasing in growth as these particles are effective in filling pores, enhancing the rate of hydration by acting as nucleation sites, increasing the amount of calcium-silicate-hydrate (C-S-H), and reducing the size of Ca(OH)_2_ crystals [21]. Many studies have proven that the compressive strength, flexural strength, tensile strength, and impermeability can be enhanced by the addition of nano materials [22,23]. Moreover, microstructure analysis shows that the nano materials improve the microstructure of cement matrix by reducing the total porosity, refining the pore structure, and decreasing the water absorption of mortar [23,24,25]. Therefore, attempts were also made to improve the damping capability of concrete prepared with recycled coarse aggregate by Liang et al. [26] and Zou et al. [27]. Their experimental results showed that the damping improvement was achieved when the concrete contained 4% of nano-SiO_2_.

Based on the literature review, there are so far fewer studies on the properties of RFA composites with GO, and the potential of GO in improving the dynamic mechanical properties of RFA composites at different temperatures has not been investigated. As important performance indicators of materials, dynamic mechanical properties are critical to the structural dynamic response, materials damage, and damping energy consumption. Currently, most of the techniques aim to increase the damping properties of structures by installing with various damping devices, such as machines or hydraulic pressure systems in order to reduce the vibration of structures. However, if the fundamental damping ratio of traditional reinforced concrete structures can be enhanced, saying, for example, from original 5% to 10% or even to 15%, the structure without additional dampers or control devices will possess enough capacity to dissipate the vibrating energy. The motivation of this paper is to investigate the potential of GO in improving the dynamic mechanical properties of RFA composites. In the present study, the dynamic mechanical properties of RFA composites with GO were measured at controlled frequency (1 Hz) and temperatures (−50 °C to 60 °C) under dynamical load (20 N to 180 N). Other properties of RFA composites, including workability, flexural, and compressive strengths, were also evaluated. To explain the enhancing mechanisms of the RFA composites with GO, material characterization analyses using SEM (scanning electron microscopy), XRD (X-ray diffraction), MIP (mercury intrusion porosimetry), etc., were also performed.

## 2. Materials and Experimental Procedure

### 2.1. Cement and Chemical Admixture

**Figure 1:**
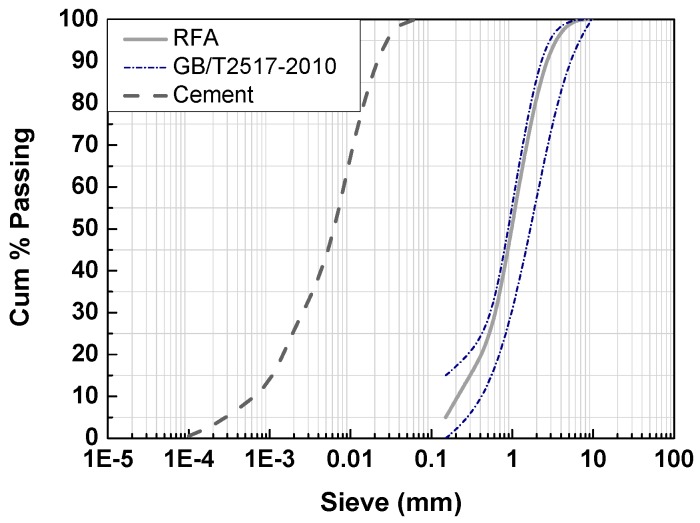
Particle-size distribution of recycled fine aggregate.

Ordinary Portland Cement (OPC) type 42.5R was used as the binder material, conforming to the requirements of Chinese Standard GB175-2007 [28]. The chemical compositions and physical properties of the cement used are shown in Table 1. The particle size of the cement was scanned by a laser particle size analyzer and its distribution is given in Figure 1.

Mixing water was normal tap water, conforming to Chinese Standard JGJ63-2006 [29]. In order to ensure the uniform dispersion of GO nanosheets, this study uses the polycarboxylate-based superplasticizer Sika TMS-YJ-1, conforming to the requirements of JG/T223-2007 [30].

### 2.2. Recycled Fine Aggregate

The recycled fine aggregate used in the experimental study reported here was obtained from a recycling plant, where mixed demolition wastes were processed by mechanical crushing, sieving, and sorting operations. All of the recycled fine aggregate was sieved first to obtain various grading sizes and the different fractions were kept in sealed containers to prevent humidity exchange with the surrounding environment. The recycled fine aggregate used is conformed to the requirements specified in GB/T25176-2010 [31], with a fineness modulus of 2.39, a mud content of 1.2%, and a clay lump content of 0.8%. The grading curve of the recycled fine aggregate is shown in Figure 1.

### 2.3. Preparation of GO

The GO (3 g/L) was prepared by dispersing the graphite oxide powder into the mixture of water and polycarboxylate ether (PCE) superplasticizer (PCE/GO ratio of 1.3) with the help of ultrasonication. Graphite oxide was purchased from the Sixth Element Ltd. (Changzhou, China). The properties of the Graphite oxide are given in Table 2. As mentioned earlier, if GO is to be fully utilized in materials, it must undergo proper dispersion. Based on previous investigations [32], the ultrasonication conditions were thus used as follows: the power 600 W, frequency 20 Hz and time 2 h, titanium alloy probe width 20 mm.

### 2.4. Characterization Methods

After ultrasonication, visual inspection of GO was carried out by employing atomic force microscope (AFM, type ICON-PT-PKG, Bruker, Fremont, CA, USA) and transmission electron microscopy (TEM, type JEM-1230, NIPPON TEKNO company, Osaka, Japan). Raman scattering was conducted on a Renishaw RM 3000 Micro-Raman system (Bruker, Fremont, CA, USA) at 1800 nm grating for 10 s. The chemical bonding of GO was measured by using Fourier transform infrared spectrometer (FTIR, type AVANCE 600 MHz, Bruker, Switzerland).

### 2.5. Mixtures Proportioning and Preparation

The focus of this work is not on the development of RFA mortar, but on the use of advanced techniques to examine the effect of GO on the dynamical mechanical properties of RFA composites. The mix proportions of various RFA composites are given in Table 3. The aqueous GO suspensions were made with the mixing water and the GO substances. Based on previous investigations and the high-water absorption rate of recycled sand, the water/cement ratio (w/c) was selected as 0.66 to ensure the adequate workability of the RFA mortar. Based on the work [33,34], the amounts of GO used in the RFA mortar were 0.00%, 0.05%, 0.10%, and 0.20% by mass of cement, respectively, while the PCE/GO ratio used was 1.3.

**Figure 2:**
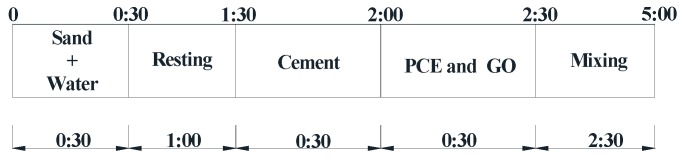
Mixing sequence of RFA mortar (unit: min).

RFA mortar was mixed in a high-shear mixer. The temperatures of the materials and the mixer were controlled at room environment. The mixing sequence of the RFA mortar includes, wetting the recycled sand followed by the addition of cement. The initial wetting of the recycled sand was carried out to ensure that the recycled sand could be coated by a layer of cement paste, which would enhance the quality of the interface between the recycled sand and hydrated cement paste. The PCE and GO diluted with the remaining mixing water were then introduced over 30 s, and then the mixture was mixed for a total time of 2.5 min, as shown in Figure 2. Without the mixing sequence of the recycled sand, the other mixing sequences of paste were the same as the RFA mortar. Once the mixing stopped, the mixture was poured into a mini-cone to carry out the fluidity measurement. After the measurement, the RFA mortars were cast into molds with dimensions of 40 mm × 40 mm × 160 mm and 40 mm × 40 mm × 40 mm for the flexural and compressive strength tests, respectively, and were then cast into the customized molds of 30 mm × 30 mm × 30 mm for the dynamic mechanical analysis.

### 2.6. Test Methods

#### 2.6.1. Workability

The effect of GO on the workability of cement paste and RFA mortar were evaluated by a mini-slump test. After preparation of cement paste and RFA mortar, the fresh mixture was poured into a mini-cone (top inside diameter: 36 mm, bottom inside diameter: 60 mm, height: 60 mm) for the fluidity evaluation. The testing method was conducted according to the requirements of GB/T2419-2005 [35].

#### 2.6.2. Flexural and Compressive Strengths

To examine the influence of GO on the mechanical properties of RFA mortar, both the compression test and three-point bending test were conducted. The size of specimens that were used for the flexural strength tests was 40 mm × 40 mm × 160 mm and that used for the compressive strength test was 40 mm × 40 mm × 40 mm. These specimens were tested at 14 days and 28 days. Three specimens were repeated in both the flexural and compressive strength tests. The tests were carried out via a computerized electronic universal testing machine (YZH-300, Shenzhen Kedao experimental equipment Co., Ltd., Shenzhen, China) with loading rate of 20 N/s and 2.4 KN/s, respectively, for the flexural and compressive tests. The strength tests are in accordance with the standard of GB/T17671-1999 [36].

#### 2.6.3. DMA

The dynamic mechanical properties of materials are often measured by using the dynamic mechanical analyzer (DMA) [37,38]. The elastic (or storage) modulus, viscous (or loss) modulus, and loss factor can be obtained from DMA testing; their relationships are given as follows:

(1) $$E^{\prime }=\sigma_{0}\cos\delta /\varepsilon_{0}\;E^{\prime \prime }=\sigma_{0}\sin\delta /\varepsilon_{0}$$

**Figure 3:**
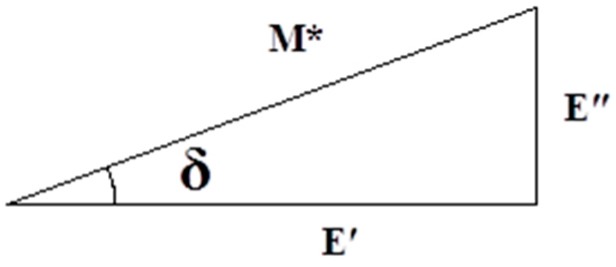
Relationship between the loss angle and modulus.

(2) tanδ=12πΔww=E″E′

(3) $$\Delta w={{\pi \varepsilon }_{0}}^{2}\;E^{\prime \prime }$$

(4) $$w=\frac{1}{2}{\varepsilon_{0}}^{2}\;E^{\prime }$$

(5) $$M^{*}=E^{\prime }\left(1+i\;\tan\delta \right)$$

where E′ is the elastic modulus or storage modulus, E″ is the viscous modulus or loss modulus, σ_0_ is the stress, ∆w is as the material vibrates the energy consumed in a cycle, w is the maximum strain energy stored by the material for one cycle, ε_0_ is the strain, and δ is the phase angle, M* is the complex modulus. Figure 3 shows the relationship among the storage modulus, loss modulus, composite modulus and loss angle.

**Figure 4:**
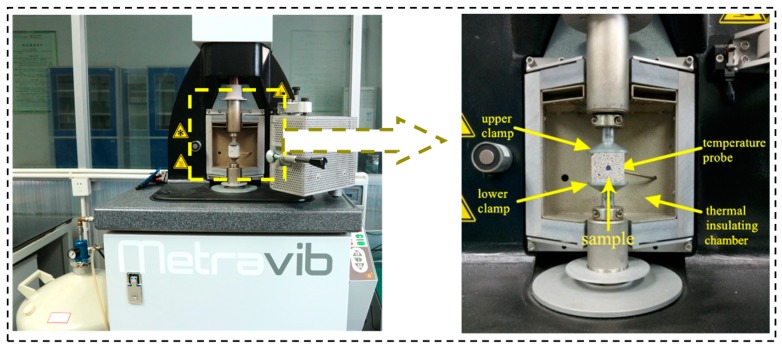
Test setup of dynamic mechanical analyzer (DMA).

In this study, the dynamic mechanical property test was carried out by using a dynamic mechanical analyzer (DMA+1000, Metravib, France) at a frequency of 1 Hz, a heating rate of 5 °C/min, temperature ranging from −50 °C to 60 °C, a maximum dynamic force of 80 N, and a static force of −100 N. Note that the dynamic properties of RFA mortar under low frequency waves can be easily obtained by using a dynamic mechanical analyzer. The low frequency waves, in the range of 1–10 Hz for earthquakes, can cause an extensive damage, depending on the geological conditions and the matching resonant fundamental frequency of buildings [39]. The selected temperature range is the range in which most of structures serve. Figure 4 shows an image of the test set-up. The tests were carried out on the specimens at the age of 14 days and 28 days. To eliminate the effect of void water, the specimens were dried in a vacuum desiccator to a constant weight at 40 °C. Based on the repeatability of DMA tests proved by Yuan et al. [37] and Foray-Thevenin et al. [38], DMA tests were carried out on the same sample.

#### 2.6.4. Microstructure Characterization

The element analysis of cement paste samples was carried out using XRD. Peaks in calcium hydroxide (CH), calcium-silicate-hydrate + calcium carbonate (C-S-H + CC), calcium carboaluminate hydrate + calcium hydroxide (CCA + CH), and calcium carbonate (CC) were identified in order to show the presence of hydrated cement paste. The XRD analysis was performed by using X-ray diffractometer (XRD, D8 advance, Bruker, Karlsruhe, Germany) equipped with monochromatic Cu-Ka radiation at 40 kV and 40 mA. The XRD apparatus that was employed was a continuous mode and it was used for collecting data from 5 to 800 at a scanning speed of 20/min.

The pore structure of the RFA mortar was evaluated using MIP to examine the pore size distribution. The samples were broken into 3 mm to 6 mm particles and then soaked in acetone to stop the hydration. Before MIP testing, the samples were vacuum oven-dried at 100 °C for 3 h [40] and kept in a vacuum chamber until they were tested. It should be noted that different protocols for preparing MIP specimens and conducting MIP tests could influence the results noticeably [41]. This test was performed in two steps: (i) evacuation of gasses and filling the sample holder with mercury in the low pressure of 345 kPa; and, (ii) intrusion of mercury into the sample at high pressure (maximum pressure of 420 MPa). A solid-liquid (pore wall mercury) contact angle of 130° and a mercury surface tension of 485 mN/m were used to interpret the results with the use of the Washburn equation [42,43,44].

The internal morphology of the RFA mortar was assessed by ESEM at seven days. Moreover, the fracture surface morphology after the 28-day DMA test was assessed by environmental scanning electron microscopy (ESEM, type Quanta TM 250 FEG, FEI Company, Salem, OR, USA). The plane polished sample was imaged in the backscattered mode (BSEM) to obtain high resolution micrographs that distinguishes different solid phases. Several high-resolution images were obtained at different magnifications. While the other images of samples were shot by a digital camera, all of these images were dealt with by Image J2× to obtain the volume fraction of the pores.

## 3. Results and Discussions

### 3.1. Characterization of GO

**Figure 5:**
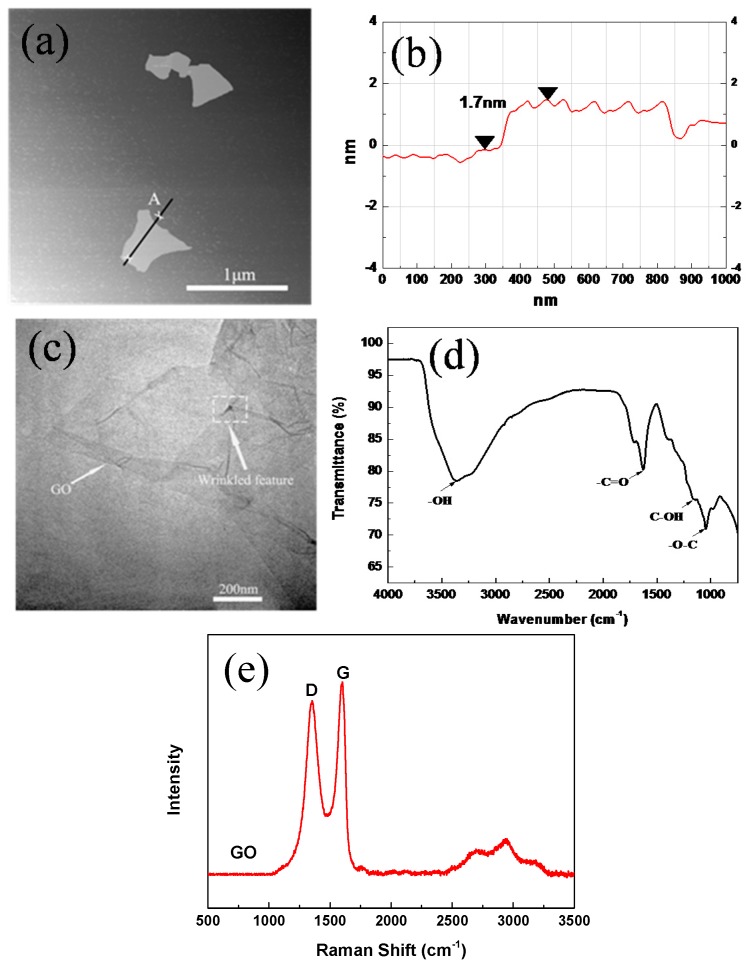
The characterization of graphene oxide (GO). (**a**,**b**) atomic force microscope (AFM) spectra of GO; (**c**) Image transmission electron microscopy (TEM) of GO; (**d**) Fourier transform infrared spectrometer (FTIR) transmittance spectra of GO; and, (**e**) Raman spectra of GO.

Figure 5a,b show the image of GO by AFM. It can be observed that GO nanosheets exhibit irregular shapes with a dimension of about 0.6 μm and a thickness of about 1.7 nm. Figure 5c shows the TEM image of GO. It can be clearly seen that GO is almost transparent nanosheets, with many wrinkled and folded features due to intercalating the oxygen-containing functional groups. These results suggested that GO was well dispersed in the mixture.

The oxygen-carbon groups of GO conformed to those of FTIR, as shown in Figure 5d. The spectrum of GO has several typical oxygen-containing characteristic peaks at 3367, 1690, and 1060 cm^−1^, which correspond to the hydroxyls (–OH), carbonyls (–C=O), and sp^3^ carbon with C–O bonds (–O–C), respectively. These oxygen functionalities endow GO with a high hydrophilicity, thus making it easily dispersed in the aqueous cement paste. GO possess a high surface area of 161 m^2^/g, which provides a significantly high contact area with the cementitious material. The elemental compositions of GO consist of 49.6 wt % C, 2.1 wt % H and 48.3 wt % O.

Figure 5e shows the Raman spectrum of GO. There are two main Raman shifts that are characterized by carbon nano-materials ranging from 1200 cm^−1^ to 1700 cm^−1^. In this context, the first band at 1620 cm^−1^ can be attributed to the graphite mode (G band), while the second band at 1380 cm^−1^ was attributed to diamondoid mode (D band) [45]. In comparison with graphite, the D band intensity/G band intensity (ID/IG) mass ratio was observed to rise, with the presence of disordered structure in graphite arising from different functional groups in the structure [46,47].

### 3.2. Influence of GO Addition on the Workability of Paste and RFA Mortar

**Figure 6:**
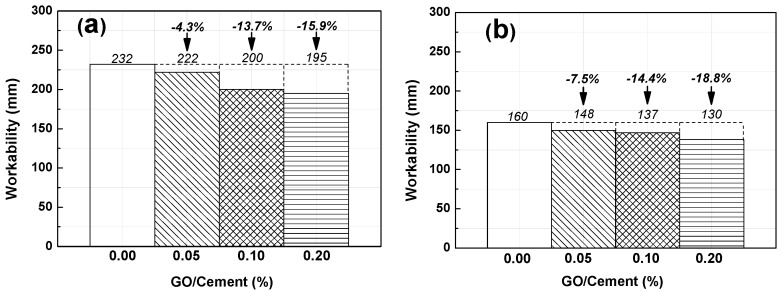
Workability results of admixture with different amount of GO: (**a**) Paste; (**b**) Mortar.

Immediately after mixing, the fresh mixture was measured by slump tests to determine the workability of the cement paste and the RFA mortar samples (R_0_, R_1_, R_2_, R_3_). The results are shown in Figure 6a for cement paste and Figure 6b for mortar; the composites with GO contents of 0.05 wt %, 0.10 wt %, and 0.20 wt %, respectively. Figure 6a shows that the workability of GO paste decreased by 4.3%, 13.7%, and 15.9% respectively with GO contents of 0.05 wt %, 0.10 wt %, and 0.20 wt %, respectively. This may be due to the fact that two-dimensional nano-structure of GO with a large specific surface area and rich oxygen containing functional groups (carboxyl, epoxy groups and hydroxyl) results in agglomeration of cement particles and the formation of a flocculation structure [48,49,50]. Figure 6b shows similar results for the RFA mortar, in which its workability decreased by 7.5%, 14.4%, and 18.8% with GO contents of 0.05 wt %, 0.10 wt %, and 0.20 wt %, respectively. From the comparison between Figure 6a,b, it can be found that the workability of the mortar prepared with recycled fine aggregate loss about 31.0%, 33.3%, 31.5%, and 33.3% when compared to that of corresponding cement paste. This might be due to the high-water absorption of the recycled fine aggregate. As mentioned earlier, GO is a super sorbent with a large surface area (like nano SiO_2_), which results in the absorption of available free water in the early ages [51].

### 3.3. Static Mechanical Properties

**Figure 7:**
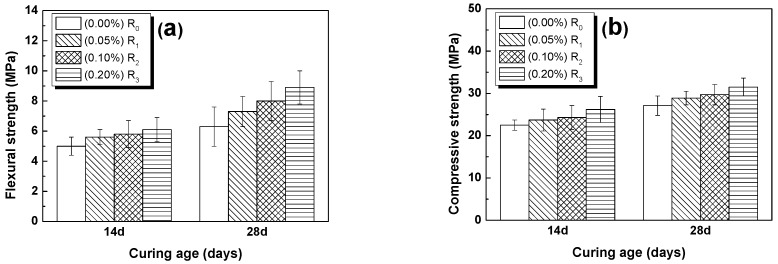
Results of strengths of RFA mortar: (**a**) flexural strengths; (**b**) compressive strengths.

The results of flexural strength tests of RFA mortar with different GO contents at different ages are shown in Figure 7a. The flexural strengths of the RFA mortar without GO were 5.0 and 6.3 MPa at 14 days and 28 days, respectively, which are in agreement with the values reported in literature [10]. The flexural strength of the RFA mortar with GO was found to be greater than that of the RFA mortar without GO. The maximum increments were 22.0% and 41.3% when the RFA mortar contained 0.2% GO at 14 days and 28 days, respectively.

The compressive strengths of samples with different amounts of GO at 14 and 28 days were plotted in Figure 7b. It is seen that the compressive strength of the mortar increased with the increase of GO added in the mortar. Also, it is shown that, the longer the cured age, the higher the compressive strength. The maximum increments of the compressive strength were 16.4% and 16.2% for the RFA mortar containing 0.2% GO at 14, and 28 days, respectively.

The results of Table 4 indicated that, under static conditions, both the flexural and compressive strengths increased with the addition of GO in the RFA mortar. As a consequence, the increase in flexural and compressive strengths suggests that the bond that is developed between GO and mortar is effective under static conditions, and thus the addition of GO has a positive impact on the process of hydration, which could directly transform into mechanical properties. In addition, GO, as a nano-scale layer material, can easily fill the pores of the cement matrix, and make the material more solid or denser. While the density of GO is low, thus, the density of cement composites with GO may not be necessarily higher than the cement composites without GO by measuring. Note that, if the matrix is denser in a cementitious material, then the mechanical properties of the material would generally be better, as demonstrated in literature [52].

### 3.4. Dynamic Mechanical Properties

The dynamic mechanical properties of the cement paste and mortar tested were characterized by the storage modulus (E’), loss factor (tan δ,) and energy absorption and dissipation [38]. The E’, tan δ, energy absorption, and dissipation of different samples are to be discussed in detail in the light of the importance of E’, tan δ, and energy in engineering applications.

#### 3.4.1. Loss Factor Analysis

The loss factor (tan δ) refers to the damping capability of materials, which can be defined as the ratio of energy dissipated as heat to the maximum energy stored in the materials. Thus, the damping capacity of the RFA mortar can be expressed by the loss factor under loading at 1 Hz. Besides, the magnitude of the loss factor is not related to the geometric factor.

**Figure 8:**
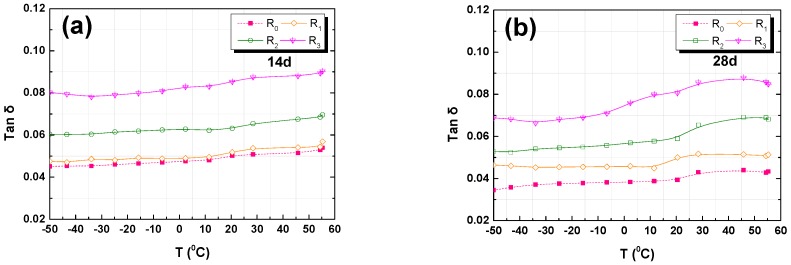
DMA analysis results of tan δ versus temperature: (**a**) 14-day; (**b**) 28-day.

The results of the tan δ analysis for the R_0_, R_1_, R_2_, and R_3_ RFA mortar specimens are shown in Figure 8a,b. It can be observed that the damping capability properties of R_0_ are different from other specimens with various GO/Cement ratios. When the w/c and sand-cement ratio (s/c) were the same, the loss factor of GO RFA mortar (R_1_, R_2_, or R_3_) was higher than RFA mortar without GO (R_0_) at the temperature between −50 °C and 60 °C; the loss factor of RFA mortar increased with the amount of added GO. The maximum increased amount of loss factor was 125% when compared to the control sample. Thus, GO addition has a positive effect on the loss factor of RFA mortar. The ability of GO to enhance the damping capability is due to its nano-material network structure, which improves the interface between individual components in the mixture, and non-uniform stress distribution under external force [27].

It is essential for the selection of damping material that is able to reach its maximum damping ability in the service environment [37]. In other words, the loss factor of RFA mortar under various temperatures needs to be considered. It was found that the curves of the loss factor of RFA mortar showed different values at various temperatures, and the tan δ increased with the increase in temperature from −50 °C to 60 °C. When comparing Figure 8a,b, it can be observed that a higher compressive strength leads to a lower loss factor for both at 14 and 28 days. The loss factor of the RFA mortar at 28 days was found to be lower than that at 14 days. It is noticed that the decrease in loss factor is corresponding to the increase in flexural and compressive strengths. This could be attributed to the porosity of the RFA mortar at 28 days, which is lower than its porosity at 14 days. This result is in good agreement with research reported by Ping [53].

#### 3.4.2. Storage Modulus Analysis

**Figure 9:**
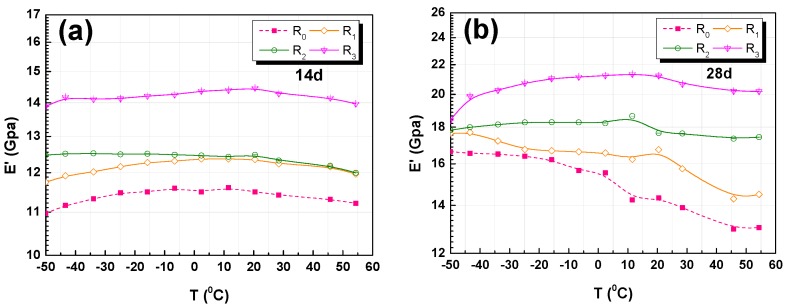
DMA analysis results of E’ versus temperature: (**a**) 14-day; and, (**b**) 28-day.

The storage modulus, which characterizes the elastic behavior of materials, is proportional to the energy that is stored by cycle. The results of the storage modulus analysis for the R_0_, R_1_, R_2_, and R_3_ RFA mortar specimens at different ages are shown in Figure 9a,b. It can be observed that the storage modulus of R_0_ RFA mortar was lower than that of the RFA mortar with GO at any temperature. The maximum increased amount of storage modulus was 53% than the control sample. Thus, GO addition is useful for storage modulus of RFA mortar. The temperature has a negative effect on the storage modulus of RFA mortar. Figure 9a,b present a larger storage modulus because of a higher compressive strength at 14, 28 days, and the storage modulus of RFA mortar is higher at 28 days than at 14 days. The increase in storage modulus is believed to be due to the strengthening of the interface between recycled fine aggregate and cement paste. In addition, the enhancement of the storage modulus by the addition of GO is due to the chemical coupling that is provided between GO and hydration products. The increase in the storage modulus is consistent with the increase in flexural and compressive strengths.

RFA mortar is a cement-based and porous composite. In this paper, the difference among R_0_, R_1_, R_2_, R_3_ lies in the content of GO added in the RFA mortar. The elastic modulus of a cement-based material is related to the elastic properties and volume fraction of the matrix (cement paste), aggregate, and the volume of void [54,55]. According to Powers and Brownyards [56], Saafi [34], and Pan [57], GO alters the morphology of geopolymers from a porous nature to a substantially pore filled morphology with increased mechanical properties. GO can improve the internal density of RFA mortar by promoting hydration reactions. Therefore, a higher GO/cement ratio leads to a lower capillary porosity and results in an increase in the volume fraction of solid phases (cement hydrate, and recycled sand). It is generally accepted that the Young’s modulus of porous materials decreases with an increasing porosity [58,59]. Therefore, as shown in Figure 9, the elastic modulus of RFA mortars increased with the increase in GO. The elastic modulus is the storage modulus. Thus, this reveals that the storage modulus of RFA mortar increases with the increase in the amount of added GO in the mixture.

#### 3.4.3. Energy Absorption and Dissipation

**Figure 10:**
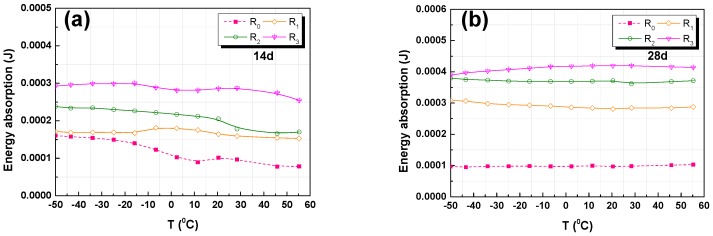
DMA analysis results of energy absorption versus temperature: (**a**) 14-day; (**b**) 28-day.

**Figure 11:**
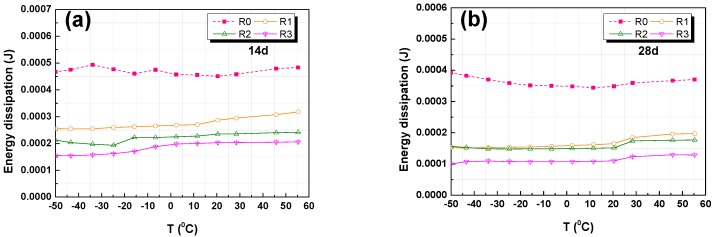
DMA analysis results of Energy dissipation versus temperature: (**a**) 14-day; and, (**b**) 28-day.

The influence of the GO addition on the energy absorption and dissipation of RFA mortar samples when subjected to temperature was examined. The values of the energy absorption and dissipation are summarized in Figure 10 and Figure 11. It is shown that, under dynamic conditions, the energy absorption of the RFA mortar significantly increased with the addition of GO, and so did the energy dissipation. This demonstrates that the addition of GO in RFA mortar has a positive effect on the energy absorption, but a negative impact on the energy dissipation.

As shown in Figure 10a,b, the increase in energy absorption with increasing contents of GO at 14 and 28 days. The total energy absorption of the addition of 0.2% GO sample R_3_ were almost twice and three times higher than the RFA mortar without GO at 14, 28 days, respectively. When comparing Figure 10a,b, it can be found that the energy absorption of RFA mortar was higher at 28 days than at 14 days. This could be explained that the GO has a positive impact on the hydration process and the pore-filling function of GO leads to an improved density of the RFA mortar. Additionally, the energy absorption increased with increasing internal products, which means that the energy absorption increased with an increasing compressive strength of the RFA mortar. The temperature had a negative effect on the energy absorption of RFA mortar at early ages but no significant change was seen in the energy absorption at 28 days. The reason for this may be due to that the internal microstructure tends to be stabilized after 28 days curing.

From the Figure 11a,b, it can be seen that the decrease in energy dissipation is along with increasing content of GO at both 14 days and 28 days. It is found that the total energy dissipation of the sample R_3_ with 0.2% GO is almost 2.5 and 3.2 times lower than RFA mortar without GO (R_0_) at 14 days and 28 days, respectively. Again, this may be due to the addition of GO, which improves the internal interface and thus leads to more energy dissipation from the RFA mortar.

### 3.5. Microstructure Characterization

#### 3.5.1. X-ray Diffraction Analysis

**Figure 12:**
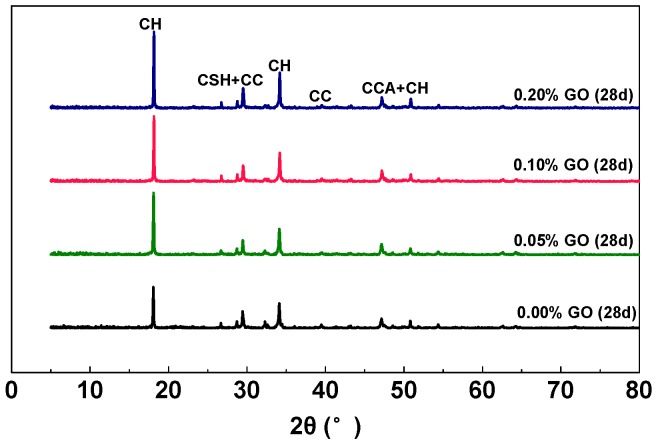
XRD patterns of different GO-cement composites at 28 days of curing. CH: Calcium hydroxide; C-S-H: Calcium silicate hydrate; CC: Calcium carbonate; CCA: Calcium carboaluminate hydrate.

Figure 12 shows the XRD patterns of the cement composites in the RFA mortar with different GO additions after 28-day of curing. The phases detected are the usual cement hydrates as C-S-H, CH and calcium carbonate (CC), and calcium carboaluminate (CCA) hydrate [60]. Based on XRD analysis, phases of CH, C-S-H + CC, and CCA + CH have the highest characteristic peaks, which are located at 2θ values of around 18°, 29°, and 48°, respectively.

Since the weight fractions of GO that were used in the RFA mortar were all rather small, there were no peaks corresponding to GO in the XRD patterns of GO-RFA mortar samples. Therefore, no major difference could be observed in the diffraction patterns of the different samples, indicating they all have similar mineralogical compositions. However, the increase of peaks was seen in the by-product of the cement hydration that was created through the reaction of the –COOH functional groups with C_2_S, C_3_S phase. The quantity of CH at early ages can be evaluated as the degree of hydration and the amount of C-S-H generation; while, the amorphous phase can be considered as the contributor to strength [61]. These mineralogical results indicate that GO can promote hydration reactions. As a result, the increase of calcium carboaluminate hydrated phase may contribute to the enhanced energy absorption and storage modulus of the RFA mortar, which is in good correlation with the results of dynamic mechanical properties analysis.

#### 3.5.2. BSEM and ESEM Microstructure Analysis

**Figure 13:**
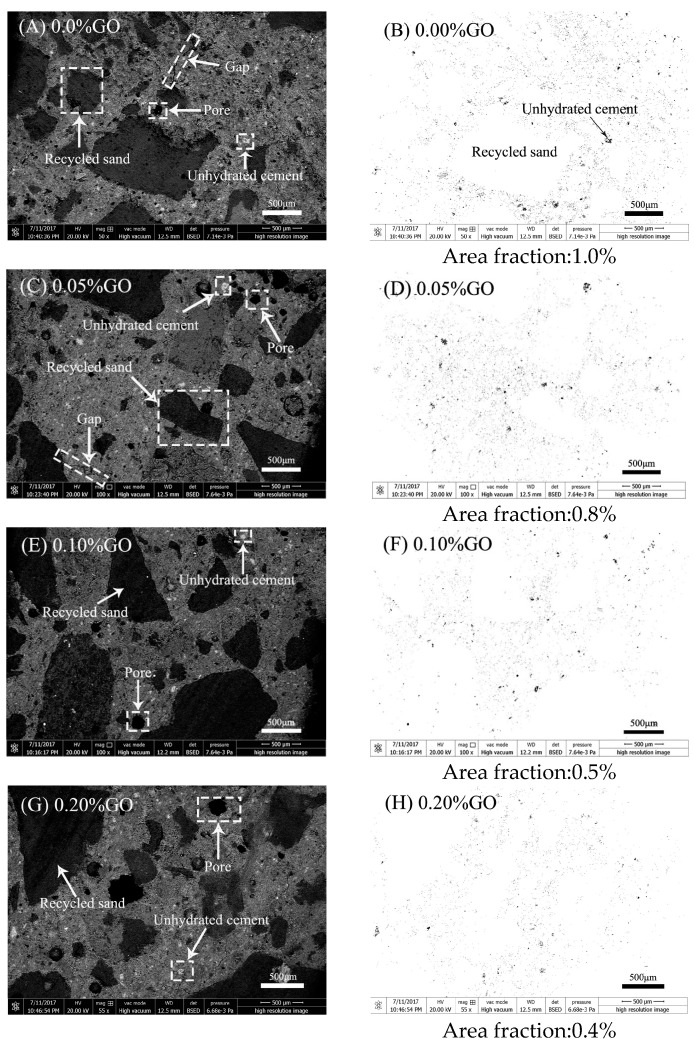
SEM and backscattered mode (BSEM) images of the area fraction of unhydrated cement at 28 days: (**A**,**B**) 0.0% GO; (**C**,**D**) 0.05% GO; (**E**,**F**) 0.10% GO; (**G**,**H**) 0.20% GO.

The microstructure characteristics of RFA mortar were analyzed using BSEM after impregnation in epoxy resin and polishing. The images of the fracture surface morphology of RFA mortar at 28 days are shown in Figure 13. As marked in Figure 13A,C,E,G, the recycled fine aggregate, pore, gap, and unhydrated cement in the RFA mortar are illustrated there. The black area shown in Figure 13B,D,F,H represents the particles of unhydrated cement in the RFA mortar, whereas the white color in the RFA mortar are the pore and recycled fine aggregate. In order to demonstrate the presence of GO affected the cement hydration, the area fraction of unhydrated cement particles was evaluated with Image J2×, and then calculated with Equation (6). The calculated area fraction of unhydrated cement particles were 1.0%, 0.8%, 0.5%, 0.4%, respectively, which corresponded to the different GO addition, 0.0%, 0.05%, 0.10%, and 0.20%.

(6) $$\text{Area fraction}=\frac{\text{Area of unhydrated cement particles}\;(\text{black area})}{\text{Total area of RFA mortar cross-section}}$$

In a previous study [62], it demonstrated that the unhydrated cement particles were associated with the degree of hydration. The addition of GO can promote the hydration of cement. Therefore, a reasonable interpretation of the BSEM images shown in the figure is the decrease of the area fraction of unhydrated cement particles when GO is added in the RFA mortar. Moreover, as the amount of GO addition increased, the area fraction of unhydrated cement particles decreased.

**Figure 14:**
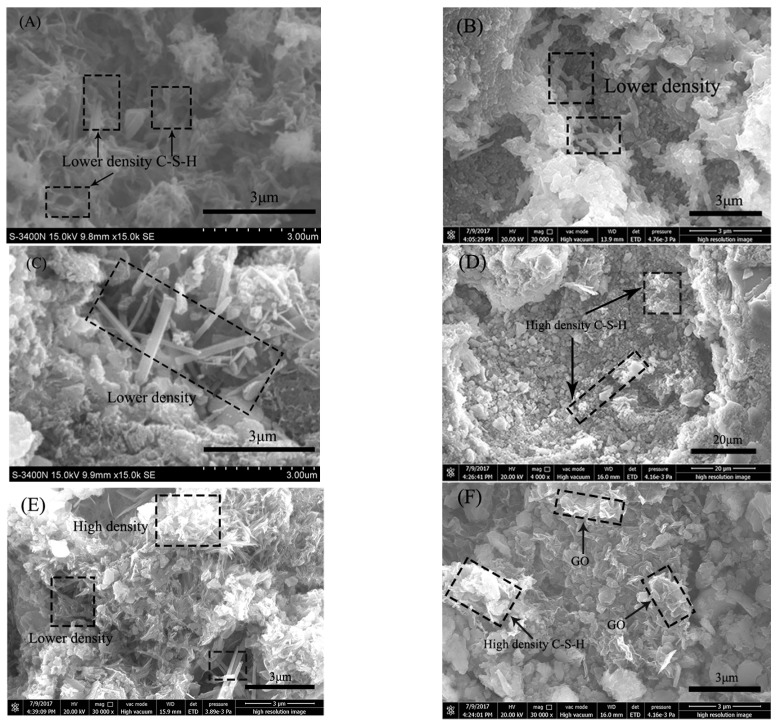
SEM images of crystal morphology at different magnifications in plain-RFA mortar sample (**A**–**C**) and in GO-RFA mortar sample (**D**–**F**) after 7 days curing.

High resolution SEM images were obtained for the RFA mortar with the different amounts of GO (0.00%, 0.05%, 0.1%, 0.2%) to examine the morphological changes at high magnification. Figure 14A–C shows the crystal morphology of RFA mortar without GO cured for 7-day. It can be seen that many pores and cracks exist in C-S-H and there are many lower C-S-H density areas. In addition, it can also be found that cracks usually pass through dense hydration products in a straight-through manner. Figure 14D–F show the crystal morphology of the RFA mortar with GO cured for 7-day. It was found that the production of thin non-uniform platelets and entangled network of rod like crystals were observed at various locations in the GO sample after 7 days. The high-density C-S-H can be seen with the addition of GO. Besides, the area of high density increased with the increase in GO content. In particular, as compared with other fillers, GO exhibits a unique two-dimensional structure, which can effectively deflect, or force cracks to tilt and twist around GO. The process may help to impede fine cracks.

The results of the microanalysis agree well with those that were examined in dynamical mechanical analysis tests. Because capillary pores, C-S-H density, cement hydrates, and sand are the main factors affecting the storage modulus of RFA mortar [63,64]. The storage modulus of the RFA mortar without GO was lower than GO-RFA mortar due to many pores and cracks in the internal microstructure.

#### 3.5.3. Pore Structure Analysis

The porosity plays an important role in influencing the mechanical properties of cement-based materials. The dynamic mechanical properties, flexural, and compressive strengths of RFA mortar are directly related to the pore structure of the materials.

**Figure 15:**
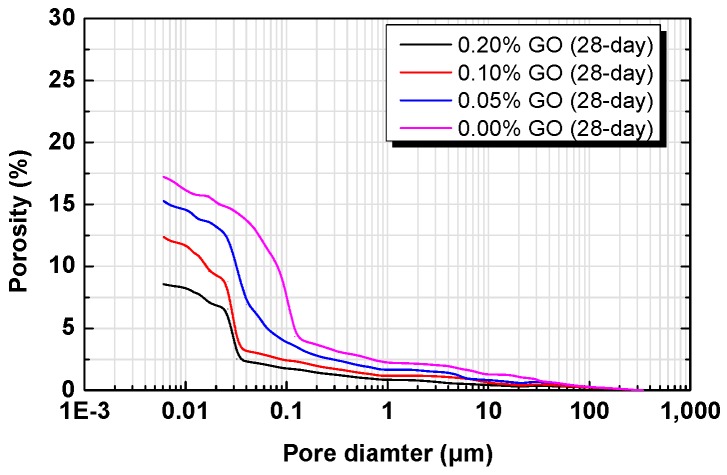
Pore size distribution of RFA mortar at 28 days.

The pore structure of the RFA mortars after 28 days was determined by MIP, and the results are represented by cumulative porosity curves, as shown in Figure 15. As it can be observed, the addition of GO in a mass ratio of 0.05%, 0.10%, and 0.20%, respectively, reduces the total porosity and the threshold pore diameter (a connectivity index) compared to the RFA mortar without GO. The total porosities of these mortar samples are in the order of R_3_ < R_2_ < R_1_ < R_0_. The porosity of R_3_ (0.20% GO) is only half of the porosity of R_0_. As pores in hardened cement-based materials can only be filled by continuously formed hydration products, the lowered porosity also means that GO can promote cement hydration. Since pores have negative effects on the mechanical performance of solid [65], the results shown here are in good agreement with the flexural and compressive strengths, as well as the dynamic mechanical properties, as displayed in Figure 7, Figure 9, Figure 10 and Figure 11, respectively. In addition, although transport properties were not tested, the remarkably lowered porosity and pore connectivity (indexed by the threshold pore diameter) in GO modified RFA mortars also indicate that the incorporation of GO can improve the impermeability [66].

**Figure 16:**
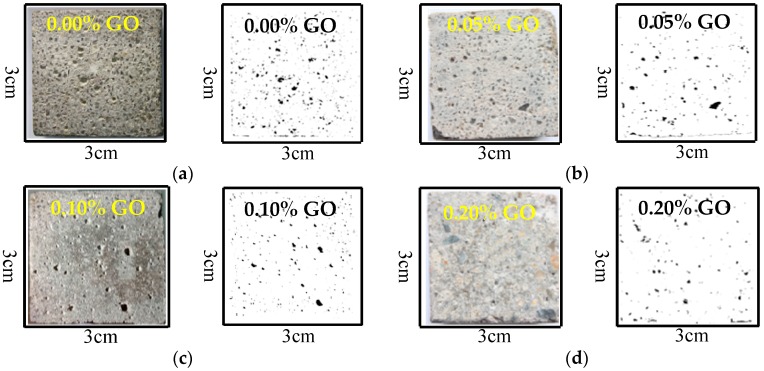
Binary images of the two-dimensional (2-D) distribution of air voids in the RFA mortars at 28 days. (**a**) Area fraction: 2.3%; (**b**) Area fraction: 1.9%; (**c**) Area fraction: 1.7%; and, (**d**) Area fraction: 1.4%.

In each column of Figure 16, the left pictures were acquired by camera, whereas the right pictures were obtained by the Image J2×. The air voids area fraction of RFA mortar is illustrated in Figure 16. The air voids area fraction of the RFA mortar without GO was 2.3%, but decreased to 1.9%, 1.7%, 1.4%, when 0.05% to 0.2% GO was added in the mortar. The maximum decrement (about 39.1%) was observed in the air voids area fraction of the RFA mortar containing 0.2% GO at 28 days. The air voids area fraction of the RFA mortar also affects the elastic properties. The elastic modulus of RFA mortar decreases with the increase in air voids content. Hence, the storage modulus of RFA mortar increases also with the addition of GO.

(7) $$\text{Area fraction}=\frac{\text{Area of pore}\;(\text{black area})}{\text{Total area of RFA mortar cross-section}}$$

## 4. Conclusions

In this study, the workability, mechanical properties, dynamical mechanical properties, and microstructure of the RFA mortar incorporating GO at different amounts of 0.05%, 0.1%, 0.2% by mass have been investigated. Based on the presented experimental results, the following conclusions can be drawn.

1. The addition of GO decreased the workability of cement paste and RFA mortar at different amounts of 0.05%, 0.1%, 0.2% by mass of cement, respectively.
2. The use of GO increased the flexural and compressive strengths of RFA mortar at 14, 28 days. The enhancement can be attributed to the accelerated cement hydration, better load-transfer efficiency, more compacted microstructure, and refined micro-cracks, owing to the existence of GO.
3. The loss factor (damping capability) of the GO-RFA mortar (R_1_, R_2_, and R_3_) was higher than that of the RFA mortar without GO (R_0_) at temperatures between −50 °C and 60 °C. The loss factor of the GO-RFA mortar increased with the increased addition of GO. The ability of GO to enhance the damping capability is due to its nano-material nature, which improves the interfaces in the mixture, and the heterogeneity of stress distribution under external force.
4. The storage modulus of R_0_ RFA mortar was lower than the other RFA mortar (R_1_, R_2_, and R_3_) with GO at any temperature. The enhancement of the storage modulus by the use of GO is due to the chemical coupling that is provided between GO and hydration products.
5. Under dynamic conditions, the energy absorption of RFA mortar is significantly increased by the addition of GO, while the energy dissipation is significantly decreased by the addition of GO. This may be attributed to the addition of GO that has a positive impact on the process of hydration and pore filling ability of the hydration products, which leads to an improved density of RFA mortar.
6. The addition of GO in cementitious materials can promote the hydration of cement. This is demonstrated by the decreased area fraction of unhydrated cement particles (BSEM) and the lowed porosity (MIP).
7. The results that were obtained from the pore structure analysis of RFA mortar (volume and surface) using MIP and Image J2x suggest that the incorporation of GO in the cement paste or RFA mortar can help in refining the pores/voids and capillary pores to a significant extent.

## Figures and Tables

**Table 1:** Chemical compositions and physical properties of cement.

**Ingredient**	**CaO**	**SiO_2_**	**Al_2_O_3_**	**Fe_2_O_3_**	**MgO**	**SO_3_**	**K_2_O**	**Na_2_O**	**LOI**
Content (mass %)	64.42	20.52	5.62	3.78	2.11	2.10	0.28	0.20	0.87
**Specific Surface Area (m^2^/g)**	**ρ^0^ (g/cm^3^)**	**Setting Time (min)**			**Stability**	**Flexural Strength (MPa)**		**Compressive Strength (MPa)**	
0.581	3.00	Initial	Final		Qualified	3 day	28 day	3 day	28 day
		112	145			6.50	9.20	34.80	58.00

**Table 2:** The properties of Graphite oxide.

Appearance	Solid Content (Mass %)	PH	Viscosity	Absorbance Ratio A230/A600	Carbon (%)	Molar Ratio (O/C)
Brown paste	43 ± 1	≥1.2	≥2000	≥45	47 ± 5	0.6 ± 1

**Table 3:** Mix proportions of paste and recycled fine aggregate (RFA) mortar.

Mix No.	Recycled Sand (g)	Cement (g)	Water (g)	w/c	GO (g)	GO/Cement	PCE/GO
R_0_	1350	450	300	0.66	0.00	0.00%	1.3
R_1_	1350	450	300	0.66	0.225	0.05%	1.3
R_2_	1350	450	300	0.66	0.45	0.10%	1.3
R_3_	1350	450	300	0.66	0.90	0.20%	1.3
P_1_	_	450	300	0.66	0.00	0.00%	1.3
P_2_	_	450	300	0.66	0.225	0.05%	1.3
P_3_	_	450	300	0.66	0.45	0.10%	1.3
P_4_	_	450	300	0.66	0.90	0.20%	1.3

Note: R is the RFA mortar, P is the cement paste.

**Table 4:** Enhancement of flexural and compressive strengths compared with R_0_.

Mix No.	No. of Specimens	Enhancement Rate of Flexural Strength (%)		Enhancement Rate of Compressive Strength (%)	
R_1_	3	+12.0 (14 day)	+15.9 (28 day)	+5.3 (14 day)	+6.6 (28 day)
R_2_	3	+16.0 (14 day)	+27.0 (28 day)	+8.0 (14 day)	+9.5 (28 day)
R_3_	3	+22.0 (14 day)	+41.3 (28 day)	+16.4 (14 day)	+16.2 (28 day)
